# Enzymatically Hydrolyzed Porcine Blood Meal as a Potential Iron Source in Canine Diets: Effects on Digestibility and Antioxidant Properties

**DOI:** 10.3390/ani16121837

**Published:** 2026-06-15

**Authors:** Yu-Jeong Na, Jun Hwang, Woo-Young Son, Eun Ju Jeong, Eui-Cheol Shin, Kyeong Soo Kim, Kwang Il Park, Ju Lan Chun, Korawan Sringarm, Chaiwat Arjin, Orranee Srinual, Hyun-Wook Kim

**Affiliations:** 1 Division of Animal Bioscience & Integrated Biotechnology, Gyeongsang National University, Jinju 52828, Republic of Korea; nayujeong2002@gmail.com (Y.-J.N.); hwangjun1116@naver.com (J.H.); sonwy001223@naver.com (W.-Y.S.); 2 Department of GreenBio Science, Gyeongsang National University, Jinju 52725, Republic of Korea; jeong.ej@gnu.ac.kr (E.J.J.); eshin@gnu.ac.kr (E.-C.S.); 3 Department of Pharmaceutical Engineering, Gyeongsang National University, Jinju 52725, Republic of Korea; soyoyu79@gnu.ac.kr; 4 Department of Veterinary Medicine, Gyeongsang National University (GNU), Jinju 52828, Republic of Korea; kipark@gnu.ac.kr; 5 Animal Welfare Division, National Institute of Animal Science, Wanju 55365, Republic of Korea; julanchun@korea.kr; 6 Department of Animal and Aquatic Sciences, Faculty of Agriculture, Chiang Mai University, Chiang Mai 50200, Thailand; korawan.s@cmu.ac.th (K.S.); chaiwat.arjin@cmu.ac.th (C.A.); orranee.s@cmu.ac.th (O.S.)

**Keywords:** canine in vitro apparent digestion, enzymatic hydrolysis, heme iron, iron fractions, porcine blood meal

## Abstract

Porcine blood meal is a protein and iron-rich animal by-product, but its use in companion animal diets is often limited by poor solubility and variable digestibility caused by thermal processing. This study evaluated whether enzymatic hydrolysis could improve the physicochemical properties, digestibility, iron-related characteristics, and antioxidant capacity of porcine blood meal for potential use in canine diets. Porcine blood meal was hydrolyzed using alcalase or pepsin under controlled conditions, and the resulting hydrolysates were characterized by degree of hydrolysis, electrophoretic peptide profiles, techno-functional properties, in vitro digestibility using a simulated canine gastrointestinal model, heme and non-heme iron fractions, and antioxidant activities. Alcalase treatment produced a higher degree of hydrolysis and more extensive peptide fragmentation than pepsin. Consistent with these structural changes, the alcalase hydrolysate exhibited significantly higher in vitro apparent digestibility. Enzymatic hydrolysis increased extractable heme iron while reducing ferrozine-reactive non-heme iron, suggesting changes in iron binding forms after proteolysis. Hydrolyzed samples also showed enhanced radical scavenging activity and ferric-reducing capacity, whereas superoxide dismutase (SOD)-like activity decreased following hydrolysis. These findings indicate that controlled enzymatic hydrolysis, particularly with alcalase, could improve apparent digestibility and non-enzymatic antioxidant capacity of porcine blood meal, supporting its potential as an iron-containing ingredient in canine diets. Further in vivo studies would be required to confirm iron availability and nutritional efficacy.

## Simple Summary

Porcine blood meal is a protein and iron-rich animal by-product of the meat industry, but its use in pet food is limited by poor solubility and variable digestibility caused by thermal processing during production. This study investigated whether enzymatic hydrolysis could improve the nutritional quality of porcine blood meal for canine diets. Hydrolysis with alcalase generated smaller peptides and significantly increased apparent digestibility under simulated canine gastrointestinal conditions. Enzymatic treatment also increased extractable heme iron and enhanced antioxidant capacity, including radical scavenging and ferric-reducing activities. These results suggest that controlled enzymatic hydrolysis may improve the nutritional and functional properties of porcine blood meal as an iron-containing ingredient in dog foods. Additional in vivo studies are necessary to confirm iron availability and nutritional benefits in dogs.

## 1. Introduction

Iron is an essential trace mineral that plays critical roles in oxygen transport, mitochondrial respiration, and cellular redox regulation in mammals [1,2]. In dogs, adequate dietary iron intake is required to maintain normal erythropoiesis, immune function, and metabolic activity [3]. The Association of American Feed Control Officials [4] recommends a minimum dietary iron concentration of 80 mg/kg on a dry matter basis for adult dogs, which corresponds to approximately 18 mg/day for a 10 kg dog [5]. Insufficient iron intake can impair oxygen transport and immune responses, whereas excessive free iron may catalyze the formation of reactive oxygen species through Fenton-type reactions, thereby contributing to oxidative stress [1,2].

In commercial companion animal diets, iron is commonly supplemented as inorganic salts such as ferrous sulfate or ferrous fumarate. Although widely used, their bioavailability may be influenced by dietary interactions, processing conditions such as extrusion, and gastrointestinal factors [6,7]. In addition, free iron ions can accelerate lipid oxidation in fat-containing feed matrices, potentially reducing product stability and palatability [8,9]. These limitations have increased interest in alternative iron sources derived from animal proteins that may provide iron in a more structurally integrated form while limiting the pro-oxidative activity of free iron.

Animal by-products have long been incorporated into pet foods as protein and mineral sources because of their high nutritional value and economic advantages [10]. Among these materials, porcine blood meal (PBM) is particularly rich in hemoglobin-derived proteins and contains substantial iron, primarily in the form of heme iron. Heme iron is generally absorbed more efficiently than non-heme iron because it is transported through specific intestinal uptake pathways that are less influenced by dietary inhibitors [11,12]. However, despite its high iron content, the use of PBM in companion animal diets remains limited. One of the primary constraints is its poor palatability, as blood-derived ingredients often exhibit a strong odor that is not readily accepted by companion animals [13]. In addition, industrial production of blood meal involves high-temperature processing and dehydration, which may induce protein denaturation, aggregation, and reduced solubility. These structural changes can negatively influence functional properties and digestibility and may also affect iron extractability and reactivity [12,14].

Enzymatic protein hydrolysis has been widely applied to improve the functional and nutritional properties of protein ingredients [15]. Proteolytic treatment generates low-molecular-weight peptides that often exhibit improved solubility and digestibility and may possess additional biological activities, including antioxidant capacity and metal binding ability [16]. In iron-containing protein systems, enzymatic hydrolysis may modify protein structures and promote the formation of peptide-associated iron complexes, which could influence iron extractability, redox behavior, and interactions within food or feed matrices [17]. Previous studies have reported the production of bioactive peptides from animal blood proteins through enzymatic hydrolysis; however, information regarding the physicochemical characteristics, iron-related properties, and digestive behavior of enzymatically hydrolyzed PBM in the context of companion animal nutrition remains limited. Based on these considerations, it could be hypothesized that enzymatic hydrolysis of porcine blood meal would improve apparent digestibility and modify iron-related characteristics by increasing extractable heme iron and altering antioxidant mechanisms.

The functional characteristics of protein hydrolysates are strongly influenced by the specificity and mode of action of the proteolytic enzyme employed. Alcalase, a broad-spectrum serine endopeptidase, has been widely reported to generate low-molecular-weight peptides with enhanced antioxidant activity and metal-chelating capacity due to its broad substrate specificity and high hydrolysis efficiency [18]. In contrast, pepsin exhibits more selective cleavage specificity under acidic conditions, producing peptides with different structural and functional properties [19]. Therefore, the use of alcalase and pepsin enables a comparative evaluation of hydrolysis patterns, allowing for clearer interpretation of differences in peptide composition, antioxidant activity, and iron binding behavior.

Therefore, the objective of this study was to evaluate the effects of enzymatic hydrolysis on the physicochemical properties, apparent digestibility, iron-related characteristics, and antioxidant capacity of porcine blood meal. PBM was hydrolyzed using alcalase and pepsin to generate hydrolysates with different peptide profiles. To assess nutritional relevance in the absence of in vivo feeding trials, a simulated canine gastrointestinal digestion model was applied. The results of this study provide insight into the potential of enzymatically hydrolyzed PBM as an iron-containing ingredient for canine diets.

## 2. Materials and Methods

### 2.1. Sample Preparation

#### 2.1.1. Raw Material

A commercially available porcine blood meal (PBM) product was purchased from the Bu-kyeong Livestock Products Auction Center (Gyeongsangnam-do, Republic of Korea). The PBM was produced from blood collected from LYD three-way crossbred pigs under standard industrial slaughter conditions. Information regarding the manufacturing process was provided by the supplier. According to the manufacturer, fresh porcine blood was collected via a closed bloodletting system during slaughter and subjected to steam heat treatment (80–90 °C) for sterilization, followed by dehydration and direct heat drying. The final dried PBM powder was vacuum packaged and stored under ambient conditions prior to use in this study. The proximate composition of porcine blood meal (PBM) was obtained from the manufacturer’s specification sheet as follows: ≥90 g/100 g crude protein, ≤0.3 g/100 g crude fat, ≤1.5 g/100 g ash, and ≤12 g/100 g moisture.

#### 2.1.2. Enzymatic Hydrolysis

PBM was dispersed in double-distilled water (DDW) at 5% (*w*/*v*) and homogenized at 10,000 rpm for 2 min. The pH was adjusted to 8.0 using 0.1 N NaOH for alcalase treatment or to 2.0 using 0.1 N HCl for pepsin treatment. Enzymatic hydrolysis was conducted using alcalase 2.4 L (EC 3.4.21.62, a declared activity of 2.4 AU/kg, density 1.18 g/mL, Novozymes, Frederiksberg, Denmark) at 50 °C or pepsin (EC 232.629.3, from porcine gastric mucosa, ≥400 units/mg, Sigma-Aldrich, St. Louis, MO, USA) at 37 °C. The enzyme-to-substrate ratio (E:S) was set at 1:100 (*w*/*w*) based on the protein content of PBM. Reactions were performed for 2 h with continuous stirring (125 rpm), as preliminary experiments confirmed that the degree of hydrolysis reached a plateau beyond this time. Enzymes were inactivated by heating at 85 °C for 20 min. The hydrolysates were cooled, filtered (500 μm mesh) to remove insoluble residues, freeze-dried, vacuum packaged, and stored at −20 °C until analysis. Three independent hydrolysis batches were prepared, and all analyses were performed using these independent replicates (*n* = 3).

### 2.2. Analysis of PBM Hydrolysates

#### 2.2.1. Degree of Hydrolysis (DH) and O-Phthaldialdehyde (OPA) Assay

The DH of PBM hydrolysates was determined in duplicate using the 10% trichloroacetic acid (TCA)-soluble nitrogen method combined with the bicinchoninic acid (BCA) protein assay [20]. Samples were mixed with an equal volume of 20% (*w*/*v*) TCA and centrifuged at 1600× *g* for 30 min. The supernatant containing TCA-soluble fraction was collected. Both total protein and TCA-soluble protein contents were quantified using a BCA protein assay kit (Thermo Scientific, Waltham, MA, USA). DH (%) was calculated as: DH (%) = (TCA-soluble protein/total protein) × 100.

The extent of protein hydrolysis was further evaluated in duplicate using the OPA assay as previously described [21]. The OPA method quantifies free α-amino groups released during proteolysis and was applied to complement the TCA-soluble nitrogen method by providing additional information on peptide bond cleavage and the formation of low-molecular-weight peptides. Briefly, sample solution was mixed with freshly prepared OPA reagent, and absorbance was measured at 340 nm after 2 min of reaction at room temperature. _L_-serine was used as the standard, and results were expressed as serine equivalents (meqv serine NH_2_/g protein).

#### 2.2.2. Protein Electrophoresis

Protein profiles were analyzed using sodium dodecyl sulfate–polyacrylamide gel electrophoresis (SDS-PAGE) as previously described [22]. Samples (30 μg protein per lane) were separated using 5% stacking and 8% separating gels and stained with Coomassie Brilliant Blue R-250. A pre-stained protein marker (10–250 kDa; Zen-Pre Stained Protein Marker, ZV-26619, Zenvia, Seoul, Republic of Korea) was used for molecular weight estimation. Low-molecular-weight peptides were further resolved using Tricine sodium dodecyl sulfate–polyacrylamide gel electrophoresis (Tricine SDS-PAGE) with 10% separating gels [23].

#### 2.2.3. In Vitro Apparent Digestibility

In vitro apparent digestibility was evaluated in duplicate using a two-step enzymatic digestion model simulating canine gastrointestinal conditions as described [24]. This model is based on previously reported in vitro digestion systems that have shown a strong correlation with in vivo apparent digestibility in dogs [24]. This model was selected to approximate gastric and small intestinal digestion relevant to companion animal feed evaluation. For the gastric phase, 2 g of sample was incubated with pepsin–HCl solution at 39 °C for 4 h. The pH was then adjusted to 7.5, and pancreatin solution was added for an additional 4 h at 39 °C to simulate intestinal digestion. Following digestion, samples were centrifuged at 3000× *g* for 10 min. The undigested residue was dried at 65 °C overnight and weighed. Apparent digestibility was calculated as: Apparent digestibility (%) = 100 – [(undigested residue weight/initial sample weight) × 100].

#### 2.2.4. Heme and Ferrozine-Reactive Non-Heme Iron Determination

Extractable heme iron was determined in duplicate according to a previously described method [25]. Samples were dissolved in water at 25 mg/mL. An aliquot (10 mL) was mixed with 20 mL acidic acetone solution (80% acetone, 18% water, and 2% HCl, *v*/*v*), homogenized at 12,000 rpm for 15 s (HG 15A, Daihan Scientific Co., Seoul, Republic of Korea), and incubated at 4 °C for 1 h in the dark. The mixture was centrifuged at 3500× *g* for 30 min and the supernatant was filtered through Whatman No. 4 paper. Absorbance was measured at 640 nm. A calibration curve was prepared using bovine hemoglobin. Heme iron was calculated from the hemoglobin equivalent using: Heme iron (mg/kg) = hemoglobin equivalent × 4 × (55.845/64,500), where 4 represents the number of iron atoms per hemoglobin molecule.

Ferrozine-reactive non-heme iron was measured in duplicate according to previously described methods [25]. Samples were dissolved in water at 25 mg/mL. An aliquot (5 mL) was mixed with 15 mL citrate–phosphate buffer (pH 5.5) and homogenized at 12,000 rpm for 15 s, followed by filtration through Whatman No. 4 paper. Filtrate (3 mL) was mixed with 1 mL of 2% (*w*/*v*) L-ascorbic acid and incubated for 20 min at room temperature. Then, 2 mL of 11.3% (*w*/*v*) TCA was added and the mixture was centrifuged at 3500× *g* for 30 min. Supernatant (3 mL) was mixed with 0.3 mL ferrozine reagent and 1.2 mL of 10% (*w*/*v*) ammonium acetate. Absorbance was measured at 562 nm (Libra S22, Biochrom, Cambridge, UK). Quantification was performed using an external calibration curve prepared from ferrous iron standards under identical conditions. Results were expressed as mg Fe per kg sample.

#### 2.2.5. Techno-Functional Properties

Water-holding capacity (WHC) and oil-absorption capacity (OAC) were determined in duplicate according to a previously described method [26]. Results were expressed as grams of retained liquid per gram of sample (g/g). Emulsifying activity index (EAI) and emulsifying stability index (ESI) were determined in duplicate following a previously described method [27]. Absorbance measurements were performed immediately after emulsion formation and after 30 min.

#### 2.2.6. Antioxidant Capacity

DPPH radical scavenging activity was determined in duplicate according to a previously described method [28]. Briefly, 100 µL of 0.1 mM DPPH solution (in ethanol) was mixed with sample solution (1 mg/mL) or DDW (control) and incubated at 25 °C for 30 min. Absorbance was measured at 515 nm, and scavenging activity was calculated as the percentage reduction in absorbance relative to the control. DPPH radical scavenging activity (%) = (A_blank_ – A_sample_)/A_blank_ × 100.

ABTS radical scavenging activity was evaluated in duplicate following the method [28]. The ABTS^+^ radical solution was generated by reacting 7 mM ABTS with 2.45 mM potassium persulfate for 16 h in the dark and diluted to an absorbance of 0.70 ± 0.02 at 734 nm. Diluted ABTS^+^ solution (5 mL) was mixed with sample aliquots and reacted for 6 min in the dark. Absorbance was measured at 734 nm, and scavenging activity was expressed as percentage inhibition relative to the blank. ABTS radical scavenging activity (%) = (A_blank_ – A_sample_)/A_blank_ × 100.

Hydroxyl radical scavenging activity was measured in duplicate according to a previously described method [29] using a Fenton reaction system. Reaction mixtures containing 1,10-phenanthroline, FeSO_4_, phosphate buffer (pH 7.4), and sample solution (5 mg/mL) were incubated with H_2_O_2_ at 37 °C for 60 min. Absorbance was measured at 515 nm using a microplate reader, and scavenging activity was calculated relative to the control. Hydroxyl radical scavenging activity (%) = (A_sample_ – A_control_)/(A_blank_ – A_control_) × 100.

Reducing power was assessed in duplicate using a modified method [30]. Sample solution (25 mg/mL) was mixed with phosphate buffer (pH 6.6) and potassium ferricyanide, incubated at 50 °C for 20 min, and then treated with trichloroacetic acid. After reaction with ferric chloride, absorbance was measured at 700 nm. Reducing power was expressed based on absorbance intensity.

Superoxide dismutase (SOD) activity was determined using a commercial OxiTec™ SOD assay kit (BIMEX Co., Ltd., Seoul, Republic of Korea) according to the manufacturer’s instructions. Samples were reacted with water-soluble tetrazolium (WST) working solution and xanthine oxidase at 37 °C, and absorbance was measured at 450 nm. SOD activity was expressed as inhibition percentage calculated from the absorbance values of the blanks and samples.

### 2.3. Statistical Analysis

All experiments were conducted using three independent hydrolysis batches prepared from separate PBM dispersions (*n* = 3). For each batch, analytical measurements were performed in duplicate as technical replicates. Data were expressed as mean ± standard deviation. Prior to statistical analysis, the assumptions of normality and homogeneity of variance were evaluated. Differences among treatments were analyzed using one-way analysis of variance (ANOVA). When significant effects were detected, mean comparisons were performed using Duncan’s multiple range test. Statistical significance was defined at *p* < 0.05. All statistical analyses were performed using SPSS statistical software (SPSS version 18.0, IBM Corp., Armonk, NY, USA).

## 3. Results and Discussion

### 3.1. Hydrolysis Characteristic

#### 3.1.1. DH and OPA Value

**Figure 1:**
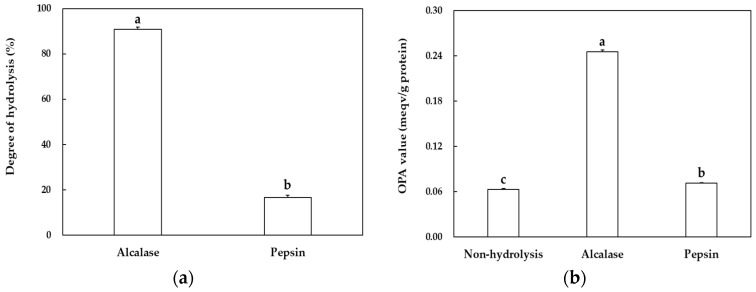
Degree of hydrolysis (**a**) and OPA-determined free α-amino group content (**b**) of porcine blood meal (PBM) hydrolysates following alcalase or pepsin treatment. Non-hydrolysis, untreated PBM control; alcalase, PBM hydrolyzed with alcalase; pepsin, PBM hydrolyzed with pepsin. Values are expressed as mean ± standard deviation (*n* = 3). a–c Different letters indicate significant differences among treatments (*p* < 0.05).

The degree of hydrolysis (DH) and OPA-determined free α-amino group contents are shown in Figure 1. Enzymatic hydrolysis significantly increased both DH and OPA values compared with the non-hydrolyzed PBM (*p* < 0.05). Among the treatments, alcalase produced the highest DH and the greatest increase in free amino groups. The higher DH observed in the alcalase-treated samples reflects the broad substrate specificity of alcalase, which acts as a serine endopeptidase capable of cleaving internal peptide bonds in diverse protein sequences [31]. In contrast, pepsin preferentially hydrolyzes peptide bonds adjacent to aromatic amino acids under acidic conditions, resulting in more limited proteolysis [32]. The agreement between DH and OPA results indicates that alcalase generated a greater extent of peptide bond cleavage and produced higher concentrations of low-molecular-weight peptides. Similar observations have been reported in other protein hydrolysis systems. For example, alcalase treatment of oilseed proteins resulted in higher DH and greater production of small peptide fractions compared with pepsin digestion [33]. Likewise, enzymatic hydrolysis of defatted peanut protein with alcalase produced a more rapid increase in DH and a pronounced shift toward smaller peptide size distributions [34]. These findings confirm that alcalase hydrolysis produced more extensive proteolytic degradation of PBM proteins than pepsin.

#### 3.1.2. Protein Electrophoresis

**Figure 2:**
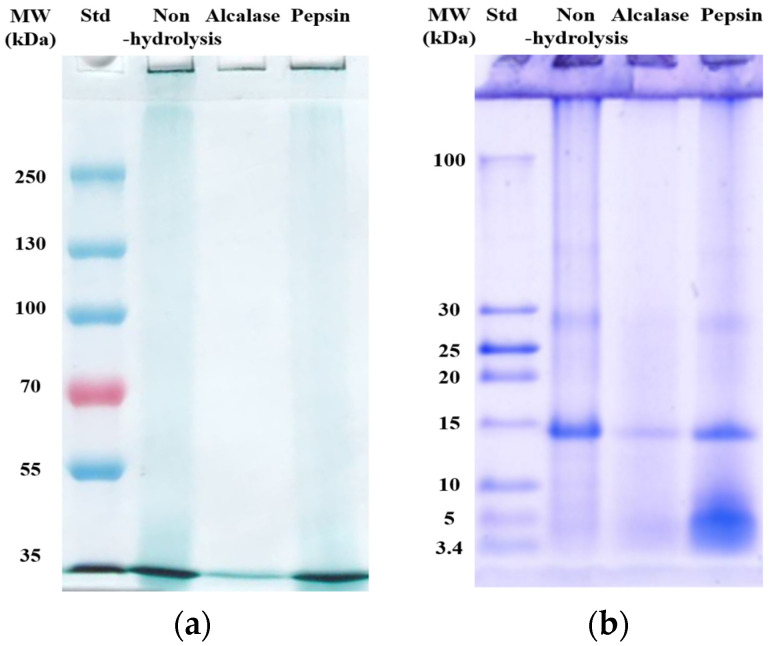
Electrophoretic profiles of porcine blood meal (PBM) before and after enzymatic hydrolysis with alcalase or pepsin. (**a**) Conventional SDS-PAGE. (**b**) Tricine SDS-PAGE for low-molecular-weight peptide resolution. Std, standard molecular weight marker; non-hydrolysis, untreated PBM control; alcalase, PBM hydrolyzed with alcalase; pepsin, PBM hydrolyzed with pepsin.

Electrophoretic patterns obtained from conventional SDS-PAGE and Tricine SDS-PAGE are presented in Figure 2. Because the resolving range of the conventional SDS-PAGE system used in this study (Figure 2a) was limited to approximately 35 kDa and above, lower molecular-weight bands could not be clearly distinguished in that system. In the non-hydrolyzed PBM, Tricine SDS-PAGE revealed a prominent band around 15 kDa (Figure 2b). This molecular weight is consistent with the reported molecular weight of hemoglobin α- and β-subunits (approximately 15–16 kDa), which are major protein components of porcine blood [35,36]. Minor diffuse staining was observed in the lower molecular-weight region of the Tricine gel, likely reflecting partial denaturation and fragmentation during industrial heat processing.

Following enzymatic hydrolysis, distinct differences in fragmentation patterns were observed between alcalase and pepsin treatments. In Tricine SDS-PAGE, the alcalase-treated sample showed marked reduction in the 15 kDa band and predominantly diffuse staining toward lower molecular weights, indicating extensive proteolytic degradation into small peptides. In contrast, the pepsin treated sample retained a visible band near 15 kDa and displayed peptide accumulation in the 3–5 kDa region, suggesting partial but more limited hydrolysis.

These results are consistent with the findings reported previously [37], which observed a similar shift in molecular weight after alcalase treatment of porcine hemoglobin. Specifically, it was reported that alcalase-induced hydrolysis led to a significant reduction in the higher-molecular-weight bands, particularly the 15 kDa band, with smaller peptides accumulating in the lower molecular weight region, similar to what was observed in this study [37]. Furthermore, pronounced degradation of protein components was also found when treated with alcalase, showing a shift towards smaller peptide fragments, which was consistent with the more extensive proteolysis observed in the alcalase-treated samples in this study [38].

Overall, these results demonstrate that alcalase induced more extensive proteolysis of PBM proteins than pepsin, generating smaller peptide fragments. The electrophoretic findings are consistent with the higher degree of hydrolysis and OPA values observed in the alcalase-treated samples and confirm the distinct peptide size distributions produced by the two enzymes.

### 3.2. In Vitro Apparent Digestibility

**Figure 3:**
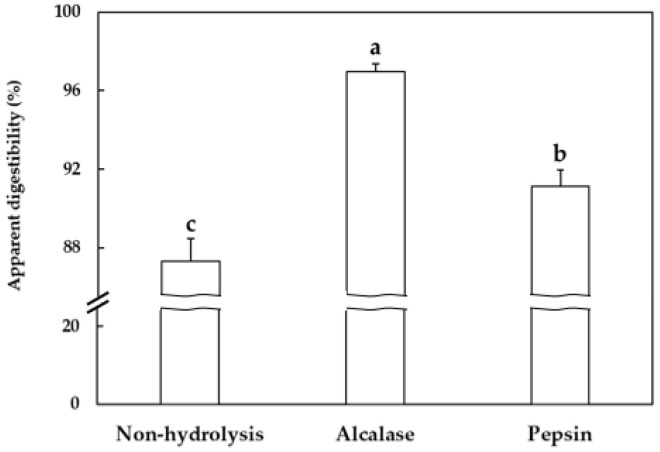
In vitro apparent digestibility of porcine blood meal (PBM) and its hydrolysates generated by alcalase and pepsin under simulated canine gastrointestinal conditions. Non-hydrolysis, untreated PBM control; alcalase, PBM hydrolyzed with alcalase; pepsin, PBM hydrolyzed with pepsin. Values are expressed as mean ± standard deviation (*n* = 3). a–c Different letters indicate significant differences among treatments (*p* < 0.05).

The in vitro apparent digestibility of PBM before and after enzymatic hydrolysis is presented in Figure 3. Enzymatic treatment significantly improved apparent digestibility compared with non-hydrolyzed PBM (*p* < 0.05). The alcalase-treated sample exhibited the highest apparent digestibility (approximately 97%), followed by the pepsin-treated sample (approximately 91%), whereas non-hydrolyzed PBM showed the lowest value (approximately 87%) (*p* < 0.05). Previous canine apparent digestibility studies have reported that the crude protein apparent digestibility of heat-processed blood meal generally ranges from approximately 75% to 85% [39,40]. In comparison, the enzymatically hydrolyzed PBM in the present study exhibited markedly higher apparent digestibility, indicating that proteolytic treatment effectively improved in vitro apparent digestibility. The marked increase in apparent digestibility following alcalase treatment is consistent with its higher DH and greater release of free amino groups. Extensive proteolytic cleavage likely reduced protein molecular size and disrupted residual heat-induced structural rigidity, thereby potentially enhancing susceptibility to simulated gastric and pancreatic enzymes [41,42]. In contrast, pepsin treatment resulted in moderate improvement, which was in line with the partial hydrolysis observed in electrophoretic analysis. Enzymatic hydrolysis has been widely reported to enhance protein apparent digestibility by reducing molecular size and improving enzyme accessibility during gastrointestinal digestion [43,44]. The correlation between proteolytic intensity (DH and OPA values) and in vitro apparent digestibility suggests that pre-hydrolysis could enhance enzymatic accessibility under simulated canine gastrointestinal conditions.

### 3.3. Heme and Ferrozine-Reactive Non-Heme Iron Fractions

**Figure 4:**
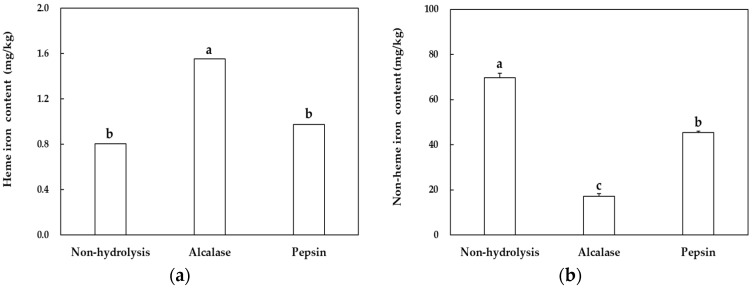
Heme (**a**) and non-heme (**b**) iron contents of porcine blood meal (PBM) hydrolysates following alcalase or pepsin treatment. Non-hydrolysis, untreated PBM control; alcalase, PBM hydrolyzed with alcalase; pepsin, PBM hydrolyzed with pepsin. Values are expressed as mean ± standard deviation (*n* = 3). a–c Different letters indicate significant differences among treatments (*p* < 0.05).

The effects of enzymatic hydrolysis on extractable heme iron and ferrozine-reactive non-heme iron are shown in Figure 4. Enzymatic hydrolysis significantly increased extractable heme iron concentrations, particularly in the alcalase-treated sample (*p* < 0.05), while ferrozine-reactive non-heme iron decreased in both hydrolysates compared with non-hydrolyzed PBM. These results suggest that enzymatic hydrolysis altered the measurable distribution of heme and ferrozine-reactive non-heme iron under the present experimental conditions. However, as these findings are based on in vitro analytical measurements, they should be interpreted with caution and do not directly reflect iron bioavailability. In addition, the measured heme and ferrozine-reactive non-heme iron fractions do not necessarily represent total iron content, as total iron was not independently determined in this study.

### 3.4. Techno-Functional Properties

#### 3.4.1. Water-Holding Capacity (WHC) and Oil-Absorption Capacity (OAC)

The techno-functional properties of PBM before and after enzymatic hydrolysis are presented in Table 1. Enzymatic treatment significantly affected WHC and OAC of PBM (*p* < 0.05). WHC decreased following hydrolysis, with the alcalase-treated sample exhibiting the lowest value. This reduction is likely attributable to proteolytic depolymerization of protein structures, since intact proteins with higher molecular weight can form three-dimensional networks that entrap water molecules [45,46]. In contrast, OAC increased in hydrolyzed samples, particularly in the alcalase-treated PBM. Proteolytic cleavage may expose hydrophobic amino acid residues that were previously embedded within the protein matrix, enhancing interactions with lipid phases [46,47]. The greater increase observed in the alcalase hydrolysate is consistent with its higher degree of hydrolysis and more extensive peptide fragmentation. These results indicate that enzymatic hydrolysis shifts PBM proteins from water-retaining network formers toward smaller peptide fractions with increased affinity for lipid binding. Enzymatic-hydrolysis-induced functional changes observed in this study were consistent with previous findings. It is reported that proteolytic treatment disrupts intact protein matrices, breaking down structured network-forming proteins and reducing their water immobilization capacity, which aligns with the decreased WHC observed in hydrolyzed PBM [48]. Previous research has further demonstrated that hydrolysis generates low-molecular-weight peptides with increased surface hydrophobicity due to the exposure of buried hydrophobic residues [46]. This structural shift corresponds with the increased OAC observed in this study, particularly in the alcalase-treated sample, indicating a transition from water retention toward enhanced lipid-binding functionality. These changes in techno-functional properties may have practical implications for pet food formulation. Reduced WHC and increased OAC could influence moisture retention, texture, and fat-binding capacity in processed products. However, the actual impact of these changes would depend on specific formulation conditions and processing systems, and further studies are required to confirm their applicability in real pet food matrices.

#### 3.4.2. Emulsifying Activity Index (EAI) and Emulsifying Stability Index (ESI)

Enzymatic hydrolysis significantly increased EAI but reduced ESI (*p* < 0.05; Table 1). The increase in EAI suggests that peptides generated during hydrolysis rapidly adsorb at the oil–water interface, enhancing initial emulsification efficiency. Smaller peptides typically exhibit improved diffusion to the interface and greater surface activity compared with intact proteins [49,50]. However, ESI decreased following hydrolysis, particularly in the alcalase-treated sample. Although small peptides facilitate rapid interfacial adsorption, the formation of a mechanically stable interfacial film requires sufficient molecular size and structural flexibility. Excessive hydrolysis may weaken interfacial film strength, resulting in reduced emulsion stability [49,51]. These findings demonstrate that enzymatic hydrolysis could improve initial emulsification capacity but compromises long-term emulsion stability. The magnitude of these changes was more pronounced in the alcalase-treated samples, reflecting their greater proteolytic intensity. These findings may be relevant for applications requiring rapid emulsification; however, reduced stability may limit their use in systems requiring long-term emulsion stability, and further validation in practical formulations is needed.

### 3.5. Antioxidant Capacity

#### 3.5.1. Radical Scavenging Activity

**Figure 5:**
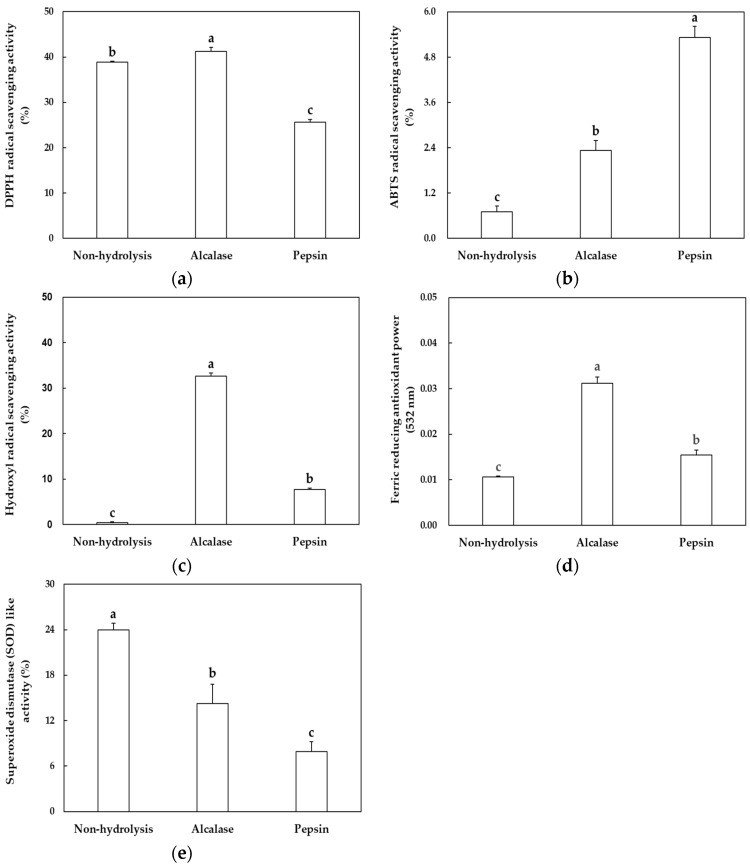
Antioxidant capacity (DPPH radical scavenging activity (**a**), ABTS radical scavenging activity (**b**), hydroxyl radical scavenging activity (**c**), ferric-reducing antioxidant power (FRAP) (**d**), and superoxide dismutase-like activity (**e**)) of porcine blood meal (PBM) hydrolysates following alcalase or pepsin treatment. Non-hydrolysis, untreated PBM control; alcalase, PBM hydrolyzed with alcalase; pepsin, PBM hydrolyzed with pepsin. Values are expressed as mean ± standard deviation (*n* = 3). a–c Different letters indicate significant differences among treatments (*p* < 0.05).

Radical scavenging activities, including DPPH, ABTS, and hydroxyl radical assays, were significantly influenced by enzymatic hydrolysis (Figure 5a–c; *p* < 0.05). Overall, hydrolyzed PBM exhibited significantly higher radical scavenging capacity than non-hydrolyzed PBM, with enzyme-dependent differences observed among assays. DPPH radical scavenging activity was highest in the alcalase hydrolysate, followed by the pepsin hydrolysate, while non-hydrolyzed PBM showed the lowest value (*p* < 0.05). The higher activity observed after hydrolysis corresponds with the increased degree of hydrolysis and OPA values. Proteolytic cleavage produces smaller peptides and exposes amino acid residues that can donate hydrogen or electrons to stabilize DPPH radicals [28]. In the ABTS assay, the pepsin hydrolysate showed slightly higher activity than the alcalase hydrolysate, although both were higher than the non-hydrolyzed control (*p* < 0.05). Differences between DPPH and ABTS results may be attributed to variations in peptide composition and polarity generated by each enzyme [28]. Hydroxyl radical scavenging activity also increased after hydrolysis. Because hydroxyl radicals are generated through iron-mediated Fenton reactions [29], the increase in scavenging activity may be associated with changes in iron binding behavior observed in hydrolyzed samples [52]. Overall, enzymatic hydrolysis could enhance radical scavenging activity, with alcalase treatment generally producing enhanced efficacies.

#### 3.5.2. Ferric-Reducing Antioxidant Power (FRAP)

Reducing power results are shown in Figure 5d. Hydrolyzed PBM exhibited significantly higher ferric-reducing capacity than non-hydrolyzed PBM, and the alcalase hydrolysate showed the greatest activity (*p* < 0.05). The ferric-reducing assay reflects the electron-donating ability of compounds present in the sample [30]. Increased reducing power after hydrolysis is consistent with the formation of low-molecular-weight peptides and exposure of reactive amino acid residues. These results agree with the higher DH and OPA values observed for the alcalase-treated samples. The enzymatic disruption of the native protein structure likely released short peptides with improved redox accessibility and exposed electron-rich amino acid residues such as histidine, tyrosine, tryptophan, and cysteine, which are known to contribute to metal ion reduction and radical scavenging [53,54]. In particular, alcalase, due to its broad substrate specificity, may have generated smaller and more mobile peptides with enhanced electron transfer efficiency compared with pepsin hydrolysates [55]. Furthermore, peptides capable of chelating ferric ions may facilitate Fe^3+^ reduction and stabilize Fe^2+^, potentially contributing to the improved iron bioaccessibility observed in this study [11,52]. Therefore, the elevated FRAP values in hydrolyzed PBM likely reflect not only enhanced antioxidant potential but also a functional role in modulating iron redox chemistry.

#### 3.5.3. Superoxide Dismutase (SOD)-like Activity

In contrast to the radical scavenging activities and FRAP, SOD-like activity decreased following enzymatic hydrolysis. Non-hydrolyzed PBM showed higher SOD activity than both hydrolysates (*p* < 0.05). Proteolytic treatment may disrupt endogenous enzyme structure, resulting in reduced catalytic activity. Structural destabilization of SOD during proteolysis has been previously reported [56,57]. These findings indicate that enzymatic hydrolysis reduced enzyme-based antioxidant activity while increasing peptide-mediated radical scavenging and reducing capacity. Similar trends have been reported in previous studies [56]. It was demonstrated that proteolytic degradation destabilizes native antioxidant enzymes such as superoxide dismutase, leading to reduced catalytic activity, and this observation was further supported in [53]. In contrast, it was observed that protease treatment enhanced peptide-mediated radical scavenging activity following hydrolysis [52]. Collectively, these findings suggest that enzymatic hydrolysis may shift antioxidant mechanisms from enzyme-dependent catalysis toward peptide-mediated radical scavenging, which is consistent with the trends observed in the present study.

## 4. Conclusions

This study demonstrated that enzymatic hydrolysis modifies the physicochemical, digestive, and antioxidant properties of porcine blood meal (PBM). Among the enzymes tested, alcalase resulted in a higher degree of hydrolysis and smaller peptide formation, which was associated with increased in vitro apparent digestibility under simulated canine gastrointestinal conditions. Enzymatic hydrolysis also altered the measurable distribution of heme and ferrozine-reactive non-heme iron. In addition, hydrolyzed PBM showed increased radical scavenging activity and ferric-reducing capacity, whereas SOD-like activity decreased following hydrolysis. These results indicate that enzymatic hydrolysis influences the functional and analytical characteristics of PBM.

However, the present findings are derived from in vitro analyses and should be interpreted with caution, as they may not fully represent iron bioavailability, antioxidant efficacy within complex food matrices, or overall in vivo nutritional outcomes. Antioxidant activity was assessed at a single concentration, and further studies incorporating multiple concentrations and EC50 determination are warranted to enable quantitative evaluation. In addition, palatability, which may influence the practical applicability of PBM in companion animal diets, was not assessed in this study. Therefore, in vivo investigations are required to validate the palatability, bioavailability, and functional relevance of hydrolyzed PBM, thereby supporting its potential application in the pet food industry.

## Figures and Tables

**Table 1:** Techno-functional properties of porcine blood meal (PBM) hydrolysates following alcalase or pepsin treatment.

Treatment	WHC (g/g)	OAC (g/g)	EAI (m^2^/g)	ESI (min)
Non-hydrolysis	1.59 ± 0.78 a	1.65 ± 0.17 b	0.01 ± 0.00 c	159.22 ± 23.58 a
Alcalase	0.28 ± 0.12 c	1.77 ± 0.18 b	0.11 ± 0.00 a	48.18 ± 2.65 b
Pepsin	0.99 ± 0.75 b	2.32 ± 0.05 a	0.07 ± 0.01 b	63.34 ± 0.77 b

WHC, water-holding capacity; OAC, oil-absorption capacity; EAI, emulsifying activity index; ESI, emulsion stability index. Non-hydrolysis, untreated PBM control; alcalase, PBM hydrolyzed with alcalase; pepsin, PBM hydrolyzed with pepsin. Values are expressed as mean ± standard deviation (*n* = 3). a–c Different letters within the same column indicate significant differences among treatments (*p* < 0.05).

## Data Availability

The data presented in this study are available on request from the corresponding author.
